# Ventricular remodeling and hemodynamic changes in heart failure patients with non-ischemic dilated cardiomyopathy following dapagliflozin initiation

**DOI:** 10.1186/s43044-024-00508-z

**Published:** 2024-06-18

**Authors:** Ahmed Hassan, Kerollos Samaan, Ahmed Asfour, Yasser Baghdady, Amir Anwar Samaan

**Affiliations:** Department of Cardiovascular Medicine, Kasr Alainy Faculty of Medicine, Cairo University, Cairo, Egypt

**Keywords:** Sodium–glucose co-transporter inhibitors (SGLT-2i), Dapagliflozin, Heart failure with reduced ejection fraction (HFrEF), Left ventricular end-diastolic pressure (LVEDP), Left ventricular extracellular volume (ECV)

## Abstract

**Background:**

In heart failure with reduced ejection fraction (HFrEF), sodium–glucose co-transporter inhibitors (SGLT-2i) have persistently shown cardiovascular benefits through different trials. However, their impact on ventricular remodeling and cardiac hemodynamics has not been sufficiently studied. This study aimed to study how SGLT-2i initiation affects invasive hemodynamics and cardiac magnetic resonance imaging (CMR)-derived ventricular volumes, function, and fraction of the extracellular volume (ECV) in HFrEF patients with non-ischemic dilated cardiomyopathy (NIDCM).

**Results:**

In this study, 23 patients with HFrEF and a mean age of 42, including 82.6% males, all have NIDCM and underwent right heart catheterization and CMR at the initiation of dapagliflozin and at 6-month follow-up. The addition of dapagliflozin resulted in significant reductions in the following invasive hemodynamic parameters compared to baseline: left ventricular end-diastolic pressure (23.4 vs 19.7 mmHg, *p* = 0.003), mean pulmonary artery pressure (31.3 vs 27.7 mmHg, *p* = 0.03), and systemic vascular resistance (18 vs 15 Wood units, *p* = 0.047). Among the studied CMR-derived measurements, only the percentage of extracellular volume fraction was significantly less at follow-up (33.7 vs 32.16%, *p* = 0.001). Additionally, functional class showed significant improvement with a notable reduction of the NT-proBNP level and a considerable decrease in diuretic dose (median: 40 vs 80 mg, *p* = 0.01).

**Conclusion:**

Adding dapagliflozin to patients with HFrEF due to NIDCM improved invasively measured hemodynamics and significantly reduced left ventricular extracellular volume fraction measured by CMR, with no significant change in ventricular volumes or ejection fraction.

## Background

Studies have consistently shown that inhibitors of sodium–glucose co-transporter 2 effectively reduce the incidence of major adverse cardiovascular events in patients diagnosed with heart failure with reduced ejection fraction (HFrEF), irrespective of their diabetes status. Dapagliflozin, similar to empagliflozin, has significantly improved clinical outcomes across a wide range of ejection fraction in patients with heart failure [1–5]. Based on these plentiful data, SGLT-2i was included in all guidelines as an integral pillar in the medical therapy of HFrEF patients.

Hemodynamic derangement is a fundamental part of heart failure pathophysiology. Limited data on the hemodynamic response to sodium–glucose co-transporter 2 inhibitors (SGLT-2i) in HFrEF are available. In the EBRACE-HF trial, SGLT-2i was associated with a significant improvement in pulmonary artery systolic pressure, as recorded by an implantable device [6]. However, a full understanding of the hemodynamic changes following the administration of SGLT-2i is still lacking.

Cardiac remolding is strongly associated with the progression of heart failure and worsening of clinical outcomes [7, 8]. Previous studies on cardiac remodeling have shown inconsistent results regarding the impact of SGLT-2i on ventricular volumes and function. However, some meta-analyses have indicated that SGLT-2i can significantly reduce cardiac volumes and improve left ventricular systolic functions [9, 10] Nevertheless, the impact of SGLT-2i on patients with non-ischemic dilated cardiomyopathy (NIDCM) has not been thoroughly studied. Furthermore, the exact mechanism by which SGLT-2i achieves these effects remains unclear [11, 12].

Cardiac magnetic resonance imaging (CMR) can reliably assess ventricular volumes and systolic function and can help assess extracellular volume (ECV), which reflects diffuse myocardial fibrosis, provided there is no myocardial edema, inflammatory processes, infiltrative diseases, or ischemia. This approach also allows for evaluation of the effect of therapeutic interventions on ECV [13]. The impact of dapagliflozin on ECV needs further studying.

The primary objective of this study was to delineate the effects of dapagliflozin on invasive hemodynamics in patients with NIDCM and evaluate its impact on ventricular volumes, function, and ECV using cardiac CMR. The present study focused only on NIDCM to avoid the confounding effect of revascularization during the study period if ischemic cardiomyopathy was included.

## Methods

The study was a single-center, prospective analytic study conducted over 18 months, starting in October 2021. It included patients known to have NIDCM who were being followed at specialized heart failure and cardiomyopathies clinics at a tertiary care facility in Egypt. The study was commenced following the ethical committee’s approval. Obtaining written consent from all participants was a prerequisite for their inclusion.

The sample size calculation was based on reference data from the results of the EMBRACE-HF trial, which evaluated empagliflozin effect on pulmonary artery pressure in patients with previously implanted pulmonary artery pressure sensors. In the sample size calculator, the study needed 21 pairs to obtain a power of 80% and a 5% significance level.

Patients with NIDCM and HFrEF NYHA class II to ambulatory IV, as defined by the ESC guidelines of heart failure, were evaluated for eligibility for this study. Patients at least 18 years old and have been on maximally tolerated doses of guidelines-directed medical therapy (GDMT) for at least 6 months, except for SGLT-2i, were included.

The study excluded newly diagnosed HFrEF patients and those with significant coronary artery disease, atrial fibrillation, very low GFR (< 30 mL/min/1.73 m), and type 1 diabetes mellitus. Patients with contraindications to CMR (e.g., claustrophobia, MRI non-compatible metallic implants) were also excluded.

The following were recorded at baseline and follow-up:

- Clinical data: basic demographics, assessment of symptoms as NYHA functional class, and clinical examination.
- Medications history: all recruited patients have been on the highest tolerated doses of GDMT for 6 months or more, except for SGLT-2 inhibitors.
- Laboratory workup: routine laboratory tests in addition to NT-proBNP were checked.
- 6-Minute walk test (6MWT): the patient was instructed to walk as much as possible within six minutes while oxygen saturation level was monitored using a portable pulse oximeter. The total walking distance and any symptoms occurred were recorded.
- A transthoracic echocardiography study was performed for each patient, including a comprehensive evaluation of LV and RV functions.

### Cardiac Magnetic Resonance Protocol

A 1.5 T AERA unit from Siemens System was utilized. Cine images using steady-state free precession (SSFP) with breath-holding were obtained to quantify the volumes and ventricular function. A gadolinium dosage of 0.15 mL per kilogram of the subject's body weight was administered to obtain delayed enhancement images, with image capture occurring 10 min post-contrast injection.

**Fig. 1:**
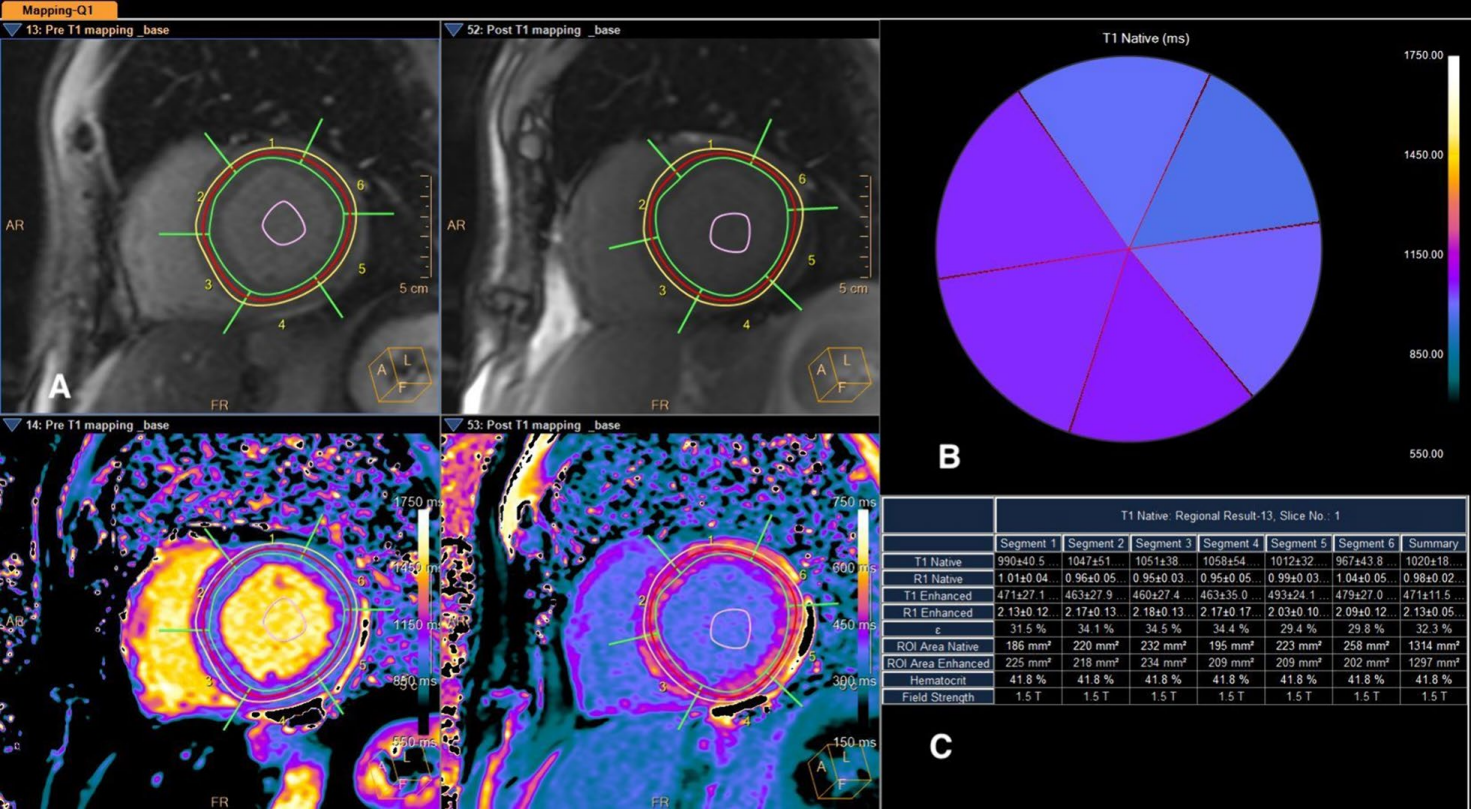
Example for calculation of ECV. **A**—Green; endocardial, Yellow; epicardial tracking, Purple; blood pool. **B**—Polar map for T1 in 6 segments basal level. **C**—Table of T1 and ECV fraction automatically calculated

In the process of T1 mapping, maps at three different levels of the heart (base, middle, and apex) were carefully outlined along the inner and outer layers of the heart muscle. These maps were then divided into 16 segments. After gadolinium contrast administration and late gadolinium enhancement (LGE) imaging, T1 mapping was conducted again 15 min after contrast injection. This was done using the same cross sections of the heart but employing a modified look-locker inversion recovery sequence (MOLLI) to evaluate each segment’s extracellular volume (ECV) fraction. The calculation of the ECV fraction, expressed in percentage, is automatically performed using the formula: ECV fraction (%) = (1 − Hematocrit) × (ΔR1myocardium/ΔR1blood). Here, R1 = (1/T1 post-gadolinium − 1/T1 pre-gadolinium) (Fig. 1).

### Right Heart Catheterization

Conventional RHCs were performed using either the right femoral or internal jugular veins. Another access through the right radial artery was used to measure the left ventricular end-diastolic pressure (LVEDP). The procedures were performed at room air. Pressures were measured, and samples were collected from each chamber. A pigtail catheter was utilized to assess the LVEDP according to the recommended technique. The position of the external pressure transducer was maintained at the mid-axillary level. The pressure measurement was repeated at least three times while checking appropriate zeroing. The LVEDP measurement was performed just before the onset of the rapid rise in the LV systolic pressure immediately after the atrial contraction phase in the pressure tracing waveform, and corresponding to the R wave in the ECG tracing. Calculations for systemic vascular resistance (SVR), pulmonary vascular resistance (PVR), cardiac index (CI), and stroke volume indexed (SVi) were then performed.

### SGLT-2 Inhibitors Initiation and Patients’ Follow-Up

Dapagliflozin 10 mg was added to the background heart failure medication. Six months after the initiation of dapagliflozin therapy, patients were re-evaluated through a comprehensive follow-up protocol for clinical assessment, transthoracic echocardiography, CMR, and RHC.

### Statistical Analysis

We utilized version 26 of SPSS software for the Mac operating system. Frequencies and percentages [*n* (%)] were used for categorical parameters. Based on the results, continuous data that underwent a normality check were displayed as mean, standard deviation, or median, interquartile range. To compare the continuous variables between the baseline and follow-up, a paired *t* test or a two-tailed Wilcoxon rank sum test was utilized based on the data distribution. A 2-sided *P* value of less than 0.05 was considered significant.

## Results

Throughout the period of the study, 31 patients with NIDCM were evaluated for eligibility. Five patients were excluded because they had atrial fibrillation, and three patients refused to participate. The remaining 23 patients were included and had baseline workup, which were repeated six months after dapagliflozin initiation.

The mean age of participants was 42.1(± 3.8) years, 19 patients (82.6%) were males, and the median duration from initial heart failure diagnosis to recruitment was 12 (interquartile range 9–20) months. Twenty patients (87%) had familial DCM. Table 1 demonstrates baseline characteristics and demographics.

**Table 1:** Baseline Characteristics and Demographics

Age (years) (Mean ± SD	42.1	(± 3.8)
Male sex (*n*) %	19	(82.6%)
Weight (Kg) (Mean ± SD)	74.9	(± 3.9)
BMI Kg/m^2^ (Mean ± SD)	25.6	(± 1.47)
BSA (Mean ± SD)	1.873	(± 0.05)
Hypertension (*n*) %	6	(26.1%)
Diabetes Mellitus (*n*) %	5	21.7%
Smoking (*n*) %	10	(43.5%)
Duration of heart failure (months)(median) (IQ range)	12	(9–20)
Diagnosis		
Familial DCM (*n*) (%)	20	(8(7%)
Post-myocarditis DCM (*n*) %	1	(0.3%)
LV non-compaction (*n*) %	1	(4.3%)
Biventricular Arrhythmogenic cardiomyopathy (*n*) %	1	(4.3%)

*BMI* Body mass Index, *BSA* Body surface area*, DCM* Dilated Cardiomyopathy, *LV* Left Ventricular

Compared to baseline, there was a significant decrease in the percentage of patients with NYHA functional class III/IV [15 (65.2%) Vs. 9 (39.1%) (*P*: 0.05)], a significant decline in NT-proBNP (2830 [775–3875] Vs. 426 [237–2045] (*P*: 0.001)) and a notable reduction in diuretic dose (80 [20–80] mg Vs. 40 [20–80] mg (*P*:0.01)). There was no statistically significant difference from baseline to follow-up in 6-min walk distance, Hematocrit, serum uric acid, creatinine clearance, doses of heart failure medications, and other echocardiographic parameters as shown in Table 2.

**Table 2:** Clinical, Laboratory, and Echocardiographic Data

	Baseline	Follow-up	*P* value
NYHA class III/IV (Number/Percentage)	15 (65.2%)	9 (39.1%)	**0.05**
Creatinine clearance ml/min/1.73m^2^	118.08 (± 39.3)	120.6 (± 43.47)	0.534
6 MWT (median-IQ range)	260 (104–390)	324 (102–400)	0.18
NT-proBNP (median-IQ range)	2830 (775–3875)	426 (237–2045)	**0.001**
Hematocrit	41.172 (± 3.78)	42.311 (± 1.17)	0.222
Serum uric acid	6.2428 (± 0.51)	5.89 (± 0.54)	0.363
Serum sodium	138.35 (± 2.8)	137.61 (± 3.71)	0.401
Serum potassium	4.35 (± 0.4)	4.29 (± 0.52)	0.666
Diuretic dose (furosemide equivalence) (median-IQ range)	80 (20–80)	40 (20–80)	**0.01**
Echocardiographic parameters			
Left ventricular ejection fraction	21.83 (± 1.821)	27.61 (± 2.71)	0.45
EDD (median-IQ range)	6.7 (6.4–7.4)	6.8 (6.1–7.6)	0.501
ESD	6.037 (± 0.733)	5.623 (± 1.2)	0.115
E/e, (median-IQ range)	15 (14–17)	11 (9–16)	**0.006**
E wave deceleration time msec (median-IQ range)	102 (67–134)	134 (100–146)	0.1
Estimated PASP (mmHg)	35 (27–56)	35 (25–40)	0.082
TAPSE mm (mean ± SD)	17 (± 5)	19 (± 5.5)	0.234
TAPSE/PASP (mean ± SD)	0.48 (± 0.05)	0.62 (± 0.71)	0.1

*6 MWT*: 6-Minute walk test, *EDD*: End-diastolic diameter, *ESD*: End-systolic diameter, *PASP*: Pulmonary artery systolic pressure, *TAPSE*: Tricuspid annulus plane systolic excursion, significant difference  (*P* < 0.05 is written in bold)

Analysis of the CMR showed a significant reduction in the average extracellular volume (ECV) fraction on follow-up compared to baseline (33.7% (± 1.3) vs. 32.2% (± 1.3). *P* = 0.001). No changes were noticed in the other CMR measurements, as illustrated in Table 3.

**Table 3:** Changes in Cardiac Magnetic Resonance Parameters During the Study Duration

	Baseline		Follow-up		*P* value
LV ejection fraction (%)	23.8	(± 9.24)	27.3	(± 13.5)	0.2
LV EDVi (ml/m2)	178.28	(± 10.962)	177.78	(± 12.060)	0.84
LV ESVi (ml/m2)	140	(± 44.29)	131.8	(± 57.73)	0.553
SVi (ml/m2)	41.22	(± 3.9)	43.5	(± 2.79)	0.72
ECV (average of 16 segments) (%)	33.74	(± 1.28)	32.16	(± 1.29)	**0.001**
CO (L/min)	5.2	(± 1.6)	5.6	(± 1.8)	0.1
RV EF (%)	38.11	(± 4.14)	40.22	(± 3.28)	0.55
RV EDVi (ml/m2) (median/range)	100	(78–111)	90	(73–127)	0.366
RV ESVi (ml/m2) (median/range)	60	(31–79)	47	(38–88)	0.831

*LV* Left ventricle, *EDVi* End-diastolic volume indexed, *ESVi* End-systolic volume indexed, *Svi* Stroke volume indexed, *ECV* Extracellular volume, *CO* Cardiac output, *RV* Right ventricle, significant difference  (*P* < 0.05 is written in bold)

On follow-up, repeated RHC revealed a significant decrease in systolic (44 [32–58] Vs 40 [31–45] mmHg *P* = 0.035) and mean pulmonary artery pressure (31.3 [ ± 10.5] Vs 27.7 [ ± 7.3] mmHg, *P* = 0.03). A significant decline was also shown in left ventricular end-diastolic pressure (23.4 [8.4] Vs. 19.7 [7.1] mmHg, *P* = 0.003) and systemic vascular resistance (18 [25–22] Vs. 15 [13–19] Wood Units, *P* = 0.047). Table 4 provides a summary of the hemodynamic parameters that were recorded at the baseline and follow-up.

**Table 4:** Changes in Right Heart Catheterization Data During the Study Duration

	Baseline		Follow-up		*P* value
RAP (mmHg) (Median + /IQ range)	10	(7–13)	8	(7–12)	0.45
PASP (mmHg)	44	(32–58)	40	(31–45)	**0.035**
PADP (mmHg)	25	(17–34)	20	(17–23)	**0.005**
mPAP (mmHg)	31.3	(± 10.52)	27.7	(± 7.32)	**0.03**
LVEDP (mmHg)	23.4	(± 8.42)	19.7	(± 7.12)	**0.003**
COP (L/min)	3.96	(± 0.92)	4.43	(± 1.51)	0.077
CI (L/min/m2)	2.09	(± 0.12)	2.34	(± 0.17)	0.104
SV (ml/m2)	48.79	(± 15.89)	54.92	(± 4.92)	0.06
SVi	26	(20–31.9)	27.7	(21.6–39)	0.072
SVR (WU)	18	(15–22)	15	(13–19)	**0.047**
PVR (WU)	2.7	(± 1.59)	2.1	(± 1.59)	0.596

*RAP* Right atrial pressure, *PASP* Pulmonary artery systolic pressure, *PADP* Pulmonary artery diastolic pressure, *mPAP* mean pulmonary artery pressure, *LVEDP* Left ventricular end-diastolic pressure, *COP* Cardiac output, *CI* Cardiac index, *SV* Stroke volume, *SVi* Stroke volume indexed, *SVR* Systemic vascular resistance, *PVR* Pulmonary vascular resistance, significant difference  (*P* < 0.05 is written in bold)

## Discussion

In this prospective single-center study, starting SGLT-2i in patients with HFrEF due to NIDCM resulted in a significant reduction of invasively measured LVEDP, pulmonary artery pressure, and systemic vascular resistance in addition to a significant decrease in LV ECV fraction by CMR with no significant change in CMR-derived LV volumes or LVEF. These results provide insights into the cardiovascular advantages of SGLT-2i.

Several theories have been suggested to explain the cardiovascular benefits associated with SGLT-2i use. Glucosuria and osmotic diuresis were once thought to be the main mechanism through which these agents improve heart failure outcomes [14]. However, as studies related to diuretics in heart failure failed to show any mortality benefit, other mechanisms had to be involved with the advantages observed with SGLT-2i. Blood pressure lowering, enhanced oxygen-carrying capacity, increased consumption of ketone bodies, weight loss, increased mitochondrial calcium, and reduced oxidative stress, inflammation, and fibrosis are all among the potential favorable actions of this class of drugs [15–17].

### SGLT-2 inhibitors & invasive hemodynamics

Our study showed that follow-up right heart catheterization revealed a significant reduction in LVEDP and SVR but no substantial change in CI. This reduction in LVEDP could be attributed to the decrease in plasma volume secondary to SGLT-2 inhibitors' diuretic effect and the reduction in SVR. Congestion with interstitial fluid accumulation is a crucial characteristic of heart failure with HFrEF and is associated with increased cardiac events. Relief of such congestion is linked to better outcomes. Congestion is commonly preceded by elevated ventricular filling pressure [18].

It has been suggested that the reduction of filling pressure by SGLT-2i could be attributed to three proposed mechanisms: reducing preload (reduction of plasma volume), enhancing contractility (change in cardiac energetics and metabolism), and reducing afterload (reduction of systemic vascular resistance) [18]. A previous study showed that SGLT-2i causes a more significant decrease in interstitial fluid rather than the intravascular volume by increasing an electrolyte-free water clearance compared to other diuretics [19]. In another study, empagliflozin reduced PCWP in patients with HFrEF compared with placebo, with no significant improvement in CI [20].

The effect of SGLT-2i on SVR is controversial and has not been well-studied yet. In contrast to our study, a small randomized clinical trial that used a placebo as a control found that empagliflozin did not significantly affect SVR in patients with type 2 diabetes. It is worth noting that this study was conducted on patients with no heart failure. The study also utilized a noninvasive pulse wave contour analysis and had a 3-month follow-up period [21].

The development of PH represents a significant milestone in the course of heart failure. There is a strong relationship between pulmonary artery pressure and clinical events in heart failure. [22]. Our study showed significant reductions in PASP, mPAP, and PADP at follow-up. This reduction in pulmonary artery pressures in our cohort could be attributed to the reduction of LVEDP. Additionally, SGLT-2 inhibitors could positively affect endothelial function by enhancing nitric oxide (NO) signaling, leading to a vasodilatory effect on the pulmonary vasculature [17, 23].

Pulmonary vascular resistance did not significantly decrease at follow-up in our study. However, it is worth mentioning that the baseline PVR was only mildly elevated (Mean 2.7 ± 1.6 WU). Our study aligns with the EMBRACE-HF trial, where empagliflozin significantly decreased PASP, MPAP, and PADP as measured by CardioMEMS. However, LVEDP or PVR was not assessed in the trial. [24].

Our study found no significant increase in invasively measured CI and SVI at follow-up. However, SGLT-2i is believed to improve cardiac energetics and contractile reserve by altering cardiac fuel utilization, increasing the consumption of ketone bodies, and enhancing the oxidation of fatty acids [25]. Additionally, it has been postulated that a reduction in blood pressure and systemic vascular resistance could potentially serve as another mechanism [26–28]. Previous studies have yielded findings that are comparable to our results [21, 29]. Omar et al. found no change in CI with SGLT-2i, but pulmonary capillary wedge pressure (PCWP) was significantly reduced compared to placebo [30].

### SGLT-2 inhibitors and ventricular remodeling

In HFrEF, adverse ventricular remodeling correlates with higher hospitalization and mortality rates. Therapeutic interventions targeting the reversal or mitigation of this process are crucial for improving clinical outcomes [31, 32].

CMR is an accurate modality to follow changes in ventricular volumes and ejection fraction. In our study, there was no significant reduction in LVEDVi. LVESVi or improvement of LVEF was measured by CMR. These findings align with those of the REFORM trial, which showed no effect of dapagliflozin on LVESV or other remodeling markers [33]. However, other studies have reported reductions in left ventricular volumes with empagliflozin, indicating reverse cardiac remodeling [20, 34]. Our findings may be linked to the inclusion of patients with very low LVEF and more extensive negative remodeling at baseline.

Right ventricular volumes and ejection fraction did not show significant improvement in our cohort. There are currently insufficient data to determine the effect of SGLT-2 inhibitors on the right ventricle. A previous study showed no significant change in the volumes and functions of both ventricles with empagliflozin administration [35].

### SGLT-2 inhibitors and extracellular volume fraction 

Cardiac MRI can quantify the extracellular myocardium volume. Recent data suggest that a high ECV fraction is associated with poor HF outcomes and a shorter hospitalization-free period [36]. Wong et al. found that in type 2 diabetes mellitus, a 3% rise in extracellular volume was linked to a 52% higher likelihood of mortality or HF hospitalization [37].

Diffuse myocardial fibrosis is a slow process of fibrotic tissue acculturation around blood vessels. Unlike focal myocardial fibrosis, it is not a consequence of cell death, and it may be reversible [13]. In our study, there was a significant reduction in ECV fraction at follow-up; this comes in agreement with some previous studies [37]. A meta-analysis of six studies has found that the use of SGLT-2i leads to a significant reduction in the ECV fraction [38]. The SUGAR DM trial showed that administration of SGLT-2i resulted in a numerically lower but not statistically significant change in ECV [39].

A range of factors can cause the reduction of extracellular volume (ECV) fraction. One of the reasons could be the SGLT-2i-induced diuresis. However, in the REFORM trial, it was observed that the percentage of ECV, despite the diuretic effect of dapagliflozin, was demonstrated by the reduction in loop diuretic doses in the Dapagliflozin arm [40]. Increased hematocrit level, an input needed for calculation of the ECV, could contribute to change in ECV fraction [41]. Nevertheless, our study did not observe any significant hematocrit value changes that were statistically significant between the baseline and follow-up assessments. SGLT-2i can exert an antifibrotic effect by signaling a pathway of nutrient deprivation with subsequent upregulation of non-selective autophagy, removing dysfunctional organelles, reducing oxidative stress, and mitigating pro-inflammatory and pro-fibrotic response [17].

### Effect of SGLT-2 inhibitors on NYHA functional class, diuretic dose, and NT-proBNP levels

Our study showed a significant decrease in symptom burden after adding SGLT-2 inhibitors, as measured by NYHA functional classification, which agrees with several previous studies [42, 43]. Our study showed a significant reduction in the diuretic dose from the baseline to follow-up. The diuresis caused by SGLT-2i may have caused this reduction. This finding is consistent with those of the REFORM trial but not with those of the DAPA-HF trial [33, 42]. Furthermore, our study observed a significant decrease in NT-proBNP levels, aligning with the outcomes of numerous prior studies [39, 44].

## Conclusion

Our prospective observational study investigated the impact of dapagliflozin initiation in HFrEF patients with NIDCM. Our results demonstrated positive hemodynamic effects, significantly reducing LVEDP, mean PAP, PADP, and SVR. Additionally, the study revealed a significant decrease in the ECV fraction in the LV as measured by CMR. The patient's functional class and NT-proBNP levels improved during the study. Overall, our study highlights the positive effects of dapagliflozin on patients with NIDCM and heart failure.

## Limitations

The study was subject to certain limitations, notably the lack of a control group. Nonetheless, it is imperative to mention that during the study's design, the ESC heart failure guidelines recommend SGLT-2i as a pillar for all patients with HFrEF. We could not withhold this benefit from the participating patients. To mitigate potential confounding factors of other background therapies, the study was designed to enroll known chronic heart failure patients who had already been on stable doses of maximally tolerated GDMT before the commencement of SGLT-2 inhibitor treatment. Another limitation is using LVEDP without PCWP measurement due to the inconsistent availability of the balloon inflation technique during the study. Although LVEDP measurement is easier and more consistent than PCWP measurement, PCWP is still considered a superior prognostic indicator in heart failure [45].

## Data Availability

Upon a reasonable request from the corresponding author, data can be provided in an anonymized form.

## Abbreviations

ARNI: Angiotensin receptor blocker/neprilysin inhibitor
BMI: Body mass index
BSA: Body surface area
CO: Cardiac output
CI: Cardiac index
CMR: Cardiac magnetic resonance imaging
DCM: Dilated cardiomyopathy
ECV: Extracellular volume
GDMT: Guidelines-directed medical therapy
HFrEF: Heart failure with reduced ejection fraction
LVESD: Left ventricular end-systolic diameter
LVEDD: Left ventricular end-diastolic diameter
LVEDP: Left ventricular end-diastolic pressure
LVEF: Left ventricular ejection fraction
LGE: Late gadolinium enhancement
MRA: Mineralocorticoid receptor antagonist
NIDCM: Non-ischemic dilated cardiomyopathy
PCWP: Pulmonary capillary wedge pressure
PASP: Pulmonary artery systolic pressure
PVR: Pulmonary vascular resistance
RHC: Right heart catheterization
SGLT-2i: Sodium–glucose co-transporter inhibitors
SVR: Systemic vascular resistance
SV: Stroke volume
Svi: Stroke volume indexed
TAPSE: Tricuspid annular plane systolic excursion
6MWT: 6-Minute walk test
